# Mental illness attitudes, service provision interest and further training preferences of clinical associates

**DOI:** 10.4102/safp.v66i1.5808

**Published:** 2024-01-23

**Authors:** Saiendhra V. Moodley, Jacqueline Wolvaardt, Christoffel Grobler

**Affiliations:** 1 School of Health Systems and Public Health, Faculty of Health Sciences, University of Pretoria, Pretoria, South Africa

**Keywords:** mental illness, mental health, clinical associates, clinician attitudes, MICA-4

## Abstract

**Background:**

Non-specialist health professionals are required to provide mental health services given the burden of disease due to mental illness. The study aimed to explore the attitudes of clinical associates towards those with mental illness as well as their interest in mental health work and additional mental health training.

**Methods:**

A cross-sectional study design was utilised. The study population consisted of clinical associates based in South Africa. An electronic questionnaire was developed that incorporated the 16-item Mental Illness Clinicians’ Attitudes version 4 scale (MICA-4), which is scored out of 96 with higher scores indicating more stigmatising attitudes. Multivariate linear regression was used to determine factors associated with the MICA-4 score.

**Results:**

The mean MICA-4 score for the 166 participants who completed all 16 questions was 37.55 (standard deviation 7.33). In multivariate analysis, the factors associated with significantly lower MICA-4 scores were falling in the 25- to 29-year-old age category and indicating that a mental health rotation formed part of the undergraduate degree. More than 80% of the participants (140/167, 83.8%) indicated an interest in mental health work. Two-thirds of the participants (111/167, 66.5%) indicated an interest in a specialisation in mental health.

**Conclusion:**

The mean MICA-4 score recorded for clinical associates indicates low stigma levels towards those with mental illness. Additionally, there is significant interest in working and training in mental health.

**Contribution:**

Training programmes should take note of the contribution of a mental health rotation to a positive attitude to mental health patients. Clinical associates’ attitudes towards mental illness together with their interest in working and training in mental health suggest that they could be more widely utilised in mental health service provision.

## Introduction

The epidemiological evidence indicates alarming levels of mental illness in South Africa. Nationally-representative data is limited with the last population-based survey that assessed multiple disorders being conducted almost 20 years ago.^1,2^ It found that the lifetime prevalence of having any of the psychiatric disorders included in the study was 30.3%, and the 12-month prevalence was 16.5%.^2^ More recent national data on depression from the National Income Dynamics Study – Coronavirus Rapid Mobile Survey reported that 29% of the respondents screened positive for depression during the period April 2021 to May 2021 with approximately 5% of the respondents screening positive for severe depressive symptoms.^3^ More than half of the respondents (52%) in that survey had been affected by significant levels of depressive symptoms during the course of the coronavirus disease 2019 (COVID-19) pandemic.^3^

Studies focussing on healthcare settings in South Africa provide an indication of the scale of the problem even prior to the COVID-19 pandemic. Kagee et al.^4^ used the Structured Interview Schedule for the Diagnostic and Statistical Manual of Mental Disorders (Research Version) in a study among individuals presenting for HIV testing at five sites in the Western Cape and reported a 19.8% prevalence of alcohol use disorder and a 14.2% prevalence of major depressive disorder.^4^ The prevalence of generalised anxiety disorder and post-traumatic stress disorder (PTSD) was 5.0% and 4.9%, respectively.^4^ A study at an antiretroviral clinic in a tertiary hospital in Tshwane, Gauteng, found a 53.8% prevalence of depression using the 20-item Centre for Epidemiological Study Depression Scale.^5^ Using the Patient Health Questionnaire-9 screening instrument, 46.2% of the participants screened positive for depression in a study conducted at a primary health care (PHC) clinic in Tshwane.^6^ In Cape Town, the Mini-International Neuropsychiatric Interview (MINI Plus) was used among recruited women attending their first antenatal visit at a clinic in a low-resource setting.^7,8^ A 22% prevalence for major depressive episode and a 23% prevalence of any anxiety disorder was found.^7,8^ The 10-item Centre for Epidemiological Study Depression Scale was used by Mokhele et al.^9^ to assess postpartum depression at midwife obstetric units in Gauteng and they reported that a quarter of women had a positive result for postpartum depression.

Non-specialist health professionals in many countries have been required to provide mental health services given the burden of disease due to mental health coupled with mental health workforce shortages.^10,11,12^ Negative and stigmatising attitudes of health professionals are likely to lead to suboptimal care of mental health patients.^13,14^ A systematic review by Vistorte et al.^15^ found that primary care physicians have stigmatising attitudes towards mental health patients. This finding appeared to be common among doctors who were older and more experienced.^15^ Their attitudes were particularly negative towards patients with schizophrenia compared to those with depression.^15^ In contrast, a recent study among non-specialist medical doctors in South Africa found that the majority of participants had a positive attitude towards mental illness.^16^ A study among PHC nurses in Addis Ababa, Ethiopia, found that 48.2% of the participants had a negative attitude towards individuals with severe mental disorders, which was associated with less than 5 years’ experience and a lack of mental health training and knowledge.^17^

The scope of practice of clinical associates (South Africa’s mid-level medical worker cadre) includes aspects of mental health assessment and management.^18^ There is evidence that they do receive some training on mental health in their undergraduate degree.^19^ Though all three universities have their mental health block in the final year of study, the duration of training varies with respect to formal lectures on campus (3 hours to 1 week) and facility-based teaching and training (2–4 weeks) as well as the extent of clinical exposure to mental health patients.^19^ It has been found that the current work of a majority of clinical associates surveyed by Moodley et al.^20^ involved in taking a mental health history, conducting a mental health examination and providing counselling to mental health patients.^20^ The clinical associate cadre is a relatively new addition to non-specialists providing mental health services in South Africa, and there is potential for them to play a bigger role.^21^ The attitudes of clinical associates towards mental illness may affect the quality of patient care and could also inform their wider utilisation in mental health services. The objectives of the study were to explore the attitudes of clinical associates towards mental illness, their interest in mental health work at different levels of care and their interest in short courses and a postgraduate qualification (specialisation) in mental health. We also assessed the factors associated with mental illness attitudes, interest in mental health work and interest in specialisation in mental health.

## Methods

### Study design

A cross-sectional study design consisting of both descriptive and analytical components was utilised.

### Study population and sampling

The study population consisted of clinical associates based in South Africa. Clinical associates qualified for less than 6 months, those that had emigrated from South Africa or those that were pursuing or had completed a medical degree were excluded from participating in the study. There was no sampling as we invited all clinical associates who were reached to participate in the study.

### Measurement tools

An electronic questionnaire was developed using the Qualtrics platform. It consisted of sections related to demographic characteristics, details of undergraduate training, mental illness attitudes, interest in mental health work and interest in further training in mental health. The questionnaire incorporated the 16-item Mental Illness Clinicians’ Attitudes scale version 4 (MICA-4).^22^ The scale is used to assess attitudes of health professionals or students in any health discipline towards mental illness and was derived from MICA version 2, which was aimed at medical students.^22,23^ The scale has been reported as having good internal consistency with Cronbach’s α of 0.72, acceptable convergent validity, good face validity and good acceptability.^22^ MICA-4 uses a 6-point Likert scale and the item scores (with most items being reversed scored) are added to give an overall score that ranges between 16 and 96.^22^ Higher scores suggest negative and stigmatising attitudes towards mental illness.^22^ The authors used an expert validation panel and cognitive interviews with five clinical associates to ensure the questions related to interest in providing mental health services and further training in mental health were appropriate.^24^ The expert validation panel consisted of three family physicians familiar with clinical associate training and three psychiatrists.^24^ The changes made as a result of expert validation were relatively minor with the interest in providing mental health services and interest in further training being divided into separate sections and a postgraduate diploma being added as an example of specialisation.^24^

### Data collection

Information regarding the survey and a link to the electronic questionnaire was distributed by the Professional Association of Clinical Associates in South Africa (PACASA) and two of the three universities that train clinical associates at undergraduate level. Social media was also used to reach additional clinical associates. After clicking on the link and confirming they met the inclusion criteria, potential participants were provided with a participant information leaflet and asked to provide electronic informed consent if they wished to participate. An incentive was offered with five participants being randomly selected to receive a R1000 gift voucher. We used the available Qualtrics functionality to prevent participants from completing the survey more than once and we ensured that the participants’ identity and contact information were not linked to the questionnaire by using an end-of-survey redirect.^25^

### Data analysis

Stata version 17 (Statacorp; http://www.stata.com) was used for data analysis. The authors calculated proportions for the demographic, employment, training and interest variables. They scored each of the MICA-4 variables from 1 to 6 with items being reverse scored where appropriate and calculated a MICA-4 score by adding these together. A mean, median and other summary statistics were calculated for the MICA-4 score. Bivariate linear regression was conducted for MICA-4 scores and demographic, employment and training variables. Variables with a *p* < 0.25 were then included in an initial multivariate linear regression model with manual backwards stepwise elimination being used to arrive at the final multivariate linear regression model. The three variables related to interest in working in mental health were aggregated to a single variable ‘interest in mental health work’. Bivariate logistic regression was conducted for this variable with MICA-4 scores and demographic, employment and training characteristics as the independent variables. Variables with a *p* < 0.25 were then included in an initial multivariate logistic regression model with manual backwards stepwise elimination being used to arrive at the final multivariate logistic regression model. A similar analysis process was followed for interest in pursuing a specialisation in mental health.

### Ethical considerations

The study was approved by the University of Pretoria Faculty of Health Sciences Research Ethics Committee (778/2020). An electronic informed consent document was provided to participants followed by an option to opt out or to continue with the survey.

## Results

### Mental Illness Clinicians’ Attitudes scale version 4 scores

Of the 216 individuals who provided consent to participate in the survey, 166 completed all questions of the MICA-4. The mean MICA-4 score was 37.55 (standard deviation [s.d.]: 7.33). The median score was 37 with a range of 22 to 61. The mean and median MICA-4 scores for various demographic and employment characteristics are shown in Table 1. Participants 25 years and older had a lower (less stigmatising) mean MICA-4 score than those 20 to 24 years of age. With respect to province of current work, participants from the Free State and Northern Cape had the lowest mean MICA-4 scores of 33.25 (s.d.: 9.21) and 34.71 (s.d. 8.88), respectively. Participants working in district municipalities had a higher (more stigmatising) mean MICA score (38.69) than those working in metropolitan municipalities (36.41) as did those working in rural areas (38.46) compared to those working in urban areas (36.94). In bivariate linear regression (Table 1), the MICA-4 score was found to be significantly associated with age and province of current work. Clinical associates aged 25 to 29 years (coefficient −3.16, 95% confidence interval [CI]: −6.03; −0.28, *p* = 0.032) had significantly lower MICA-4 scores than those aged 20 to 24 years. Those currently working in Free State province (coefficient −6.68, 95% CI: −13.02; −0.35, *p* = 0.039) had significantly lower MICA scores than those working in Eastern Cape province.

**TABLE 1:** Clinical associates’ demographic and employment characteristics and Mental Illness Clinicians’ Attitudes scale version 4 scores.

Characteristic	Frequency		Median	Range	Mean	s.d.	Bivariate linear regression		
	*n*	%					Co-efficient	95% CI	*p*
**Age (*N* = 166)**									
20–24 years	36	21.7	39.0	23–58	39.78	8.69	Ref	-	-
25–29 years	82	49.4	36.5	24–55	36.62	6.36	−3.16	−6.03; -0.28	0.032
30–34 years	39	23.5	38.0	24–61	37.69	7.82	−2.09	−5.41; 1.24	0.217
≥ 35 years	9	5.4	37.0	22–44	36.56	6.77	−3.22	−8.59; 2.14	0.237
**Gender (*N* = 166)**									
Female	118	71.1	37.0	23–61	37.90	7.44	Ref	-	-
Male	46	27.7	38.0	22–53	36.57	7.15	−1.33	−3.85; 1.19	0.298
Prefer not to say	2	1.2	40.0	38–42	40.00	2.83	2.10	−8.24; 12.44	0.689
**Province of current work (*N* = 166)**									
Eastern Cape	15	9.0	38.0	25–57	39.93	9.57	Ref	-	-
Free State	8	4.8	33.5	23–53	33.25	9.21	−6.68	−13.02; -0.35	0.039
Gauteng	85	51.2	37.0	22–61	37.12	6.93	−2.82	−6.87; 1.24	0.172
KwaZulu-Natal	12	7.2	40.0	31–53	39.17	5.77	−0.77	−6.37; 4.84	0.787
Limpopo	14	8.4	38.5	29–58	39.14	7.53	−0.79	−6.17; 4.59	0.772
Mpumalanga	14	8.4	37.5	28–49	37.00	5.26	−2.93	−8.31; 2.44	0.283
Northern Cape	7	4.2	33.0	24–47	34.71	8.88	−5.22	−11.85; 1.41	0.122
North West	10	6.0	41.0	26–52	39.30	8.14	−0.63	−6.54; 5.28	0.833
Western Cape	1	0.6	42.0	-	42.00	-	2.06	−12.89; 17.02	0.785
**Municipality of current work (*N* = 155)**									
Metropolitan	74	47.7	36.0	24–55	36.41	6.56	Ref	-	-
District	81	52.3	39.0	22–61	38.69	8.05	2.29	−0.06; 4.63	0.056
**Area of current work (*N* = 166)**									
Urban	99	59.6	37.0	22–61	36.94	7.14	Ref	-	-
Rural	67	40.4	38.0	23–58	38.46	7.57	1.52	−0.76; 3.81	0.190
**Current employer (*N* = 166)**									
Provincial Department of Health	56	33.7	38.0	24–57	37.50	6.77	Ref	-	-
Private health facility or private medical practice	50	30.1	37.0	25–58	37.58	6.81	0.08	−2.73; 2.89	0.955
Non-governmental organisation	15	9.0	42.0	22–53	39.27	9.53	1.77	−2.43; 5.96	0.407
Academic institution	33	19.9	37.0	23–61	36.94	7.87	−0.56	−3.73; 2.60	0.727
Self−employed	1	0.6	26.0	-	26.00	-	−11.5	−26.05; 3.05	0.121
Unemployed	7	4.2	35.0	29–44	35.14	5.15	−2.36	−8.14; 3.43	0.422
Other	4	2.4	43.0	34−55	43.75	9.54	6.25	−1.21; 13,71	0.100
**Work setting (*N* = 165)**									
Primary health care clinic	18	10.9	37.5	23–53	37.78	7.30	Ref	-	-
Community health centre	11	6.7	36.0	22–58	39.00	10.36	1.22	−4.34; 6.78	0.665
District hospital	39	23.6	38.0	24–57	38.08	7.21	0.30	−3.84; 4.44	0.887
Regional hospital	3	1.8	29.0	29–38	32.00	5.20	−5.78	−14.84; 3.29	0.210
Tertiary or central hospital	5	3.0	42.0	32–53	41.40	7.67	3.62	−3.73; 10.97	0.332
Private general practice	30	18.2	37.5	24–55	37.83	8.02	0.56	−4.28; 4.39	0.980
Private specialist practice	4	2.4	36.0	32−37	35.25	2.36	−2.53	−10.56; 5.51	0.535
Private hospital	13	7.9	35.0	25–41	35.00	4.81	−2.78	−8.07; 2.51	0.301
Academic institution	20	12.1	38.0	25–61	37.85	7.87	0.07	−4.65; 4.79	0.976
Unemployed	8	4.9	35.0	29–44	34.88	5.49	−2.90	−9.08; 3.27	0.355
Other	14	8.5	40.0	26–48	38.86	6.62	1.08	−4.10; 6.26	0.681

s.d., standard deviation; CI, confidence interval.

Participants who completed their Bachelor of Medicine in Clinical Practice (BMCP) degrees at Walter Sisulu University had a higher (more stigmatising) MICA-4 mean score (41.19) than those that had completed their Bachelor of Clinical Medical Practice (BCMP) degrees at the University of Pretoria (37.24) and University of the Witwatersrand (36.84) (Table 2). The mean MICA-4 score for those who indicated they had a mental health rotation during their degree was 37.10 (s.d.: 7.05) compared to 43.33 (s.d.: 8.66) for those who indicated they did not. There appeared to be a trend of mean MICA-4 scores decreasing with length of the rotation. In bivariate linear regression, the MICA-4 score was found to be significantly associated with university, having a mental health rotation, length of mental health rotation and the site of the mental health rotation. Clinical associates who completed their BMCP degree at Walter Sisulu University (co-efficient 3.96, 95% CI: 0.39; 7.52, *p* = 0.030) had significantly higher MICA-4 scores than those who graduated from the University of Pretoria. Those who had indicated that they had a mental health rotation (co-efficient −6.23, 95% CI: −10.47; −1.99, *p* = 0.004) had significantly lower MICA-4 scored than those who indicated they had not. MICA-4 scores were significantly lower with a rotation length of 3–4 weeks (co-efficient −6.12, 95% CI: −10.51; −1.72, *p* = 0.07), 5–6 weeks (co-efficient −6.78, 95% CI: −11.63; −1.94, *p* = 0.006) and 7–8 weeks (co-efficient −9.46, 95% CI: −15.96; −2.96, *p* = 0.005) using no rotation as a reference. Mental Illness Clinicians’ Attitudes scale version 4 scores were also significantly lower with a rotation at a district hospital (co-efficient −6.40, 95% CI: −10.84; −1.96, *p* = 0.005), regional hospital (co-efficient −7.57, 95% CI: −12.13; −3.00, *p* = 0.001) and specialised psychiatric hospital (co-efficient −8.83, 95% CI: −16.98; −0.68, *p* = 0.034) using no rotation as a reference.

**TABLE 2:** Clinical associates’ training characteristics and Mental Illness Clinicians’ Attitudes scale version 4 scores.

Characteristic (*N*)	Frequency		Median	Range	Mean	s.d.	Bivariate linear regression		
	*n*	%					Co-efficient	95% CI	*p*
**University (*N* = 166)**									
University of Pretoria	68	41.0	39.0	22–58	37.24	7.51	Ref	-	-
University of the Witwatersrand	77	46.4	36.0	23–55	36.84	6.22	−0.39	−2.76; 1.99	0.746
Walter Sisulu University	21	12.7	41.0	26–61	41.19	9.60	3.96	0.39; 7.52	0.030
**Length of time since qualifying (*N* = 166)**									
Less than 3 years	36	21.7	38.5	23–57	37.94	7.65	Ref	-	-
Between 3 and 6 years	81	48.8	37.0	25–58	37.11	6.87	−0.83	−3.74; 2.08	0.573
More than 6 years	49	29.5	37.0	22–61	38.00	7.92	0.06	−3.14; 3.25	0.973
**Received training on assessment of patients with mental illness (*N* = 166)**									
No	2	1.2	41.5	41–42	41.50	0.71	Ref	-	-
Yes	164	98.8	37.0	22–61	37.51	7.36	−3.99	−14.30; 6.31	0.445
**Received training on management of patients with mental illness (*N* = 166)**									
No	3	1.8	41.0	27–42	36.67	8.39	Ref	-	-
Yes	163	98.2	37.0	22–61	37.57	7.34	0.90	−7.55; 9.36	0.833
**Mental health rotation formed part of the degree (*N* = 166)**									
No	12	7.2	43.0	26–57	43.33	8.66	Ref	-	-
Yes	154	92.8	37.0	22–61	37.10	7.05	−6.23	−10.47; -1.99	0.004
**Length of mental health rotation (*N* = 165)**									
No rotation	12	7.3	43.0	26–57	43.33	8.66	Ref	-	-
1–2 weeks	25	15.2	37.0	24–61	38.44	7.36	−4.89	−9.89; 0.11	0.055
3–4 weeks	83	50.3	37.0	23–58	37.22	6.88	−6.12	−10.51; -1.72	0.007
5–6 weeks	31	18.8	37.0	24–55	36.55	6.95	−6.78	−11.63; -1.94	0.006
7–8 weeks	8	4.8	34.0	22–44	33.88	7.24	−9.46	−15.96; -2.96	0.005
More than 8 weeks	6	3.6	35.0	25–53	36.33	9.46	−7.00	−14.12; 0.12	0.054
**Site of mental health rotation (*N* = 166)**									
No rotation	12	7.2	43.0	26–57	43.33	8.66	Ref	-	-
Primary health care clinic	2	1.2	39.5	31–48	39.50	12.02	−3.83	−14.61; 6.95	0.484
Community health centre	0	0.0	-	-	-	-	-	-	-
District hospital only	64	38.6	37.0	23–61	36.94	6.77	−6.40	−10.84; -1.96	0.005
Regional hospital only	47	28.3	36.0	22–53	35.77	6.78	−7.57	−12.13; -3.00	0.001
Tertiary or central hospital	21	12.7	39.0	29–58	39.81	7.49	−3.52	−8.63; 1.58	0.175
Specialised psychiatric hospital	4	2.4	34.5	27–42	34.50	6.14	−8.83	−16.98; -0.68	0.034
Combination of two or more of the above sites	16	9.6	38.5	25–53	38.50	7.82	−4.83	−10.22; 0.56	0.078
**Additional mental health training since qualifying (*N* = 166)**									
Yes	19	11.4	38.0	23–53	37.00	7.48	Ref	-	-
No	147	88.6	37.0	22–61	37.63	7.33	−0.62	−4.16; 2.91	0.727

s.d., standard deviation; CI, confidence interval.

The variables included in the initial multivariate linear regression model based on *p* < 0.25 were age, province of current work, municipality of current work, area of current work, current employer, current work setting, university, rotation in mental health, rotation length and rotation site. The final multivariate linear regression model is shown in Table 3. Clinical associates that were 25- to 29-years-old had significantly lower MICA-4 scores than those of other ages as did those who had indicated they had a mental health rotation during their clinical associate degree compared to those that indicated they had not. Those who selected ‘other’ as their current employer had significantly higher MICA-4 scores than the other options listed.

**TABLE 3:** Multivariate linear regression model of characteristics associated with the Mental Illness Clinicians’ Attitudes scale version 4 scores.

Characteristic	Co-efficient	95% confidence interval	*p*
**Age**			
25–29 years	−2.20	−4.37, −0.03	0.047
**Current employer**			
Other	7.42	0.34, 14.49	0.040
**Mental health rotation**			
Yes	−6.63	−10.82, −2.45	0.002

### Interest in working in mental health

The majority of participants were interested in working in mental health (Table 4). Three quarters of participants (*n* = 125, 75.3%) were interested in doing mental health work (strongly agree/agree) at a PHC clinic. Fewer participants indicated they were interested in working in a 72-h observation unit in a district hospital (*n* = 116, 69.9%) or in a specialised psychiatric hospital (*n* = 109, 65.3%). A total of 140 participants (83.8%) indicated an interest in working in mental health in any of the options provided. In bivariate analysis, the only variable that was significantly associated with an interest in mental health work was the MICA-4 score (odds ratio [OR]: 0.93, 95% CI: 0.88–0.99, *p* = 0.020). The other variables with *p* < 0.25 that were included in the initial multivariate model were province of current work, work setting, area of current work, length of time since qualifying, received training on assessment of patients with mental illness, length of mental health rotation and site of mental health rotation. Following stepwise elimination, only two variables remained in the final multivariate model, namely, MICA-4 score and province of current work. An increase in MICA-4 score (more stigmatising attitude) significantly reduced the odds of interest in mental health work (OR: 0.93, 95% CI: 0.88–0.99, *p* = 0.029) as did working in North West province (OR: 0.16, 95% CI: 0.04–0.64, *p* = 0.09).

**TABLE 4:** Clinical associates’ interest in working in mental health and additional mental health training.

Item	Strongly disagree		Disagree		Neither disagree nor agree		Agree		Strongly agree	
	*n*	%	*n*	%	*n*	%	*n*	%	*n*	%
**Interest in working in mental health**										
I would be interested in doing mental health work at a PHC clinic (*N* = 166)	9	5.4	10	6.0	22	13.3	48	28.9	77	46.4
I would be interested in working in a 72-h psychiatric observation unit in a district hospital (*N* = 166)	9	5.4	17	10.2	24	14.5	49	29.5	67	40.4
I would be interested in working in a specialised psychiatric hospital (*N* = 167)	15	9.0	16	9.6	27	16.2	50	29.9	59	35.3
**Interest in additional mental health training**										
I would be interested in receiving additional training in mental health in the form of short courses (*N* = 167)	13	7.8	1	0.6	4	2.4	30	18.0	119	71.3
I would be interested in pursuing a specialisation in mental health (e.g. 1- or 2-year honours degree or postgraduate diploma) (*N* = 167)	14	8.4	11	6.6	31	18.6	27	16.2	84	50.3

PHC, primary health care.

### Interest in additional mental health training

Almost 90% of the participants (*n* = 149, 89.2%) indicated an interest (agree/strongly agree) in mental health short courses (Table 4). Two-thirds of the participants (*n* = 111, 66.5%) indicated an interest in pursuing a specialisation in mental health. In bivariate analysis, interest in pursuing a specialisation in mental health was significantly associated with work setting and university where they studied. Clinical associates working in a private general practice (OR: 0.11, 95% CI: 0.13–0.94, *p* = 0.044), private hospitals (OR: 0.08, 95% CI: 0.01–0.78, *p* = 0.029) and those who indicated ‘other’ (OR: 0.05, 95% CI: 0.01–049, *p* = 0.010) had significantly lower odds of being interested in pursuing a specialisation in mental health than those working in a primary health care clinic. Clinical associates trained at the University of the Witwatersrand had significantly lower odds (OR: 0.37, 95% CI: 0.18–0.77, *p* = 0.008) of interest in a specialisation in mental health than those trained at the University of Pretoria. The other variables with a *p* < 0.25 that were included in the initial multivariate model were MICA-4 score, gender, province of current work, municipality, area of current work, rotation as part of the degree, length of mental health rotation and site of mental health rotation. Following stepwise elimination, the only variable that remained significantly associated with an interest in pursuing a specialisation in mental health was the university where the clinical associate degree was completed with clinical associates trained at the University of the Witwatersrand having lower odds of being interested in pursuing a specialisation in mental health (OR: 0.44, 95% CI: 0.23–0.85, *p* = 0.015) than those trained elsewhere.

## Discussion

The study aimed to determine the attitudes of clinical associates in South Africa towards working with those with mental illness. We used the MICA-4 scale that has been widely used globally to determine health professionals’ attitudes towards mental illness. Scored out of 96, a higher score indicates more stigmatising attitudes towards mental illness.^22^ The mean MICA-4 score of 37.55 (s.d.: 7.33) for clinical associates in our study is one of the lower scores among international studies that have used this scale indicating generally positive, or less stigmatising, attitudes. Mean MICA-4 scores above 50 have been reported by Eissa et al.^26^ among medical residents and house officers in Egypt with scores of 51.0 (s.d.: 8.7) and 51.28 (s.d.: 8.2), respectively.^26^ Several studies have reported mean MICA-4 scores above 40. A study in Baroda, India, among non-psychiatric consultants and residents at a tertiary hospital reported a mean MICA-4 score of 46 (s.d.: 9).^27^ Ghuloum et al.^28^ reported a mean MICA-4 score per question of 2.87 for nurses and 2.55 for doctors in Qatar, which equates to overall scores of approximately 46 and 41, respectively. A study in Saudi Arabia among tertiary hospital doctors reported a mean overall MICA score of 45.75 (s.d.: 7.54).^29^ An online survey among doctors in Poland found a mean MICA-score of 40.26 (s.d.: 8.93).^30^ At the lower end of the spectrum, a study among primary care doctors in four Latin American countries (Brazil, Bolivia, Chile, Cuba) reported a mean MICA-4 score of 36.3 (s.d.: 8.3) with no significant differences between countries.^31^ A similar mean MICA-4 score of 36.31 (s.d.: 7.60) was found among midwives in Ireland.^32^

The MICA-4 scale has also been used in studies in sub-Saharan Africa and in South Africa specifically. An extremely high mean MICA-4 score (67.70) was found in a study that included both clinical and non-clinical departments at a Nigerian university and teaching hospital.^33^ A study among PHC nurses in Addis Ababa, Ethiopia, also found a high mean MICA-4 score of 58 relative to other studies.^17^ A recent study among nurses working at PHC facilities in a metropolitan municipality in South Africa reported a mean MICA-4 score of 40.68 (s.d.: 9.70).^34^ Unsurprisingly, Eksteen et al.^35^ reported a low mean MICA-4 score of 32.7 among psychiatrists in South Africa. There is a lack of data on attitudes towards those with mental illness among mid-level medical workers (such as clinical associates, physician assistants, clinical officers, physician associates, etc.) and an absence of studies that have used the MICA-4 scale in these cadres. However, the mean MICA-4 score for clinical associates in our study compares well to other health professionals both in Africa and in globally.

With respect to demographic and employment characteristics, we found that MICA-4 scores were only significantly associated with the age category 25 to 29 years and ‘other’ employment. It is not clear why clinical associates in this age group would have lower MICA-4 scores than both their younger (20 to 24 years) and older colleagues (30 to 34 years). It should be noted that the mean MICA-4 score for all age categories was lower than the mean MICA-4 score for 20 to 24-year-olds. In other words, very young clinical associates seem to have more stigmatising attitudes towards mental illness than those in their mid-20s and older. This finding is in contrast to the study among nurses in South Africa, which found that MICA-4 scores increased with age.^34^ Studies among doctors in Latin America and Saudi Arabia that considered age as an independent variable did not find any association with MICA-4 scores.^29,31^ Clinical associates in the ‘other’ category were those not employed by the main employers of clinical associates in the country. Given the diverse nature of the ‘other’ employers, it is not possible to interpret this finding.

While clinical associates trained at Walter Sisulu University had higher MICA-4 scores than their counterparts trained at the University of Pretoria and University of the Witwatersrand, the mean score of 41.19 is still at the lower end of the spectrum. In addition, no association was found between MICA-4 scores and university on multivariate analysis. One possible explanation for this could be that Walter Sisulu University participants in the current study included a disproportionate number of the clinical associates that indicated they did not have a mental health rotation as MICA-4 scores were significantly associated with whether participants indicated that a mental rotation had formed part of their undergraduate clinical associate degree. Those participants who indicated that a mental health rotation formed part of their training had significantly lower MICA-4 scores. The curricula of all three universities that offer clinical associate undergraduate degrees include a mental health rotation but at two of the universities the practical training in mental health has been previously been found to some extent to happen by chance.^19^ It is possible that those who indicated they did not have a mental health rotation either did not have any contact with patients with mental health conditions in the rotation, that is, minimal exposure or could not recall the experience. There is some evidence that mental health exposure during training results in less stigmatising mental illness attitudes. A number of international studies that have measured medical students’ attitudes towards mental illness before and after a psychiatry rotation have found improvement in attitudes following a rotation when compared to a baseline.^36,37,38,39^ A study at Stellenbosch University using the Attitudes to Mental Illness Questionnaire found a significant improvement following the psychiatric rotation of fifth and sixth year medical students.^40^ Using the Mental Illness Clinical Attitudes Version 2 scale (MICA-2), Eksteen et al.^35^ reported a MICA-2 score of 43.9 in fifth year medical students prior to any psychiatry training compared to a mean score of 42.0 in sixth year medical students who had completed their psychiatry training.

There was substantial interest among study participants for additional training in mental health in the form of short courses. This finding may reflect both an interest in mental health as well as an acknowledgement of gaps in their undergraduate training. A number of gaps in the undergraduate mental health training of clinical associates have been identified in a study that explored the mental health curricula content of the three undergraduate programmes in South Africa.^19^ The duration of mental health training at undergraduate level has also been raised as a reason why short courses may be necessary.^21^ Short courses in mental health may also increase the confidence of employers in allowing more active involvement of clinical associates in the mental health service provision at their institutions.^21^

The interest in a specialisation in mental health in the current study was much higher than anticipated with almost two-thirds of the participants indicating they would be interested. While this may reflect a genuine interest in specialisation in mental health among a large proportion of clinical associates, there may be other explanations for this. It is possible the survey attracted clinical associates who had a pre-existing interest in mental health and the interest in the overall clinical associate population would be lower. The absence of career-pathing for clinical associates and the availability of only one Honours degree option in emergency medicine may have predisposed the participants to showing interest in any specialisation opportunity.^21^ We found that clinical associates trained at the University of the Witwatersrand had significantly lower odds of being interested in pursuing a specialisation in mental health. The three training programmes have different approaches to mental health including types of sites used for training, duration of training and levels of practical exposure to mental health patients.^19^ These approaches may have some influence on interest in pursuing a specialisation in mental health.^41^ It is possible that adequate exposure to a discipline may actually create more certainty that an individual does not want to pursue it as a specialisation. More students from the University of the Witwatersrand may have been aware of an alternative opportunity, namely, the Honours degree in emergency medicine as it is only offered at that institution.^42^ This knowledge may have influenced their interest in a mental health specialisation.

More than 80% of participants showed interest in working in mental health at various levels of the health system with mental health work at PHC clinics being the most popular option. Given that clinical associates are generally trained at district hospitals^43^ and the majority of posts for them in the public sector are at district hospitals, this is an interesting finding as it suggests a desire to contribute at PHC facilities in mental health and perhaps more broadly. While working at specialised psychiatric hospitals was the least popular of the options provided, the interest was still considerable at 65.2%. An increase in the MICA-4 score significantly reduced the odds of interest in mental health work as did working in North West province. It is unsurprising that participants with more stigmatising attitudes towards mental illness were less likely to be interested in mental health work. It is not readily apparent why working in North West province in particular would be linked to less of an interest in mental health work. There may be local issues with respect to mental health service provision that impacts interest in wanting to work in the field.

### Limitations

The authors tried to reach as many clinical associates as possible through their professional organisation, alumni databases and social media. However, it is not clear how many were eventually reached and how representative they were of the clinical associate population in South Africa. Incentives were offered to encourage participation, but it is possible that a proportion of participants did so because of a pre-existing interest in mental health and would have more positive mental illness attitudes than those that chose not to resulting in selection bias. Nevertheless, the crude numbers alone in this study provide sufficient evidence for interest in mental health work and additional training to make these viable options. The questionnaire used in the study was relatively lengthy and therefore a number of the participants did not complete the questionnaire. The smaller than expected initial number of participants in the study exacerbated by this drop off did affect the statistical power with respect to the inferential statistics (linear and logistic regression). Despite this, the authors were able to find some significant associations.

## Conclusion

The mean MICA-4 score recorded for clinical associates in this study is among the lowest recorded among non-specialist health professionals indicating low mental illness stigma levels. Clinical associates’ attitudes towards mental illness coupled with their interest in working and training in mental health suggests that they could be more widely utilised in mental health service provision. There appears to be willingness among clinical associates to attend short courses in mental health, which is an option to close the gaps that exist in undergraduate training. A postgraduate specialisation in mental health for clinical associates is likely to attract considerable interest should it be offered in the future.
